# Epidemiological trends and economic burden of genital warts in Dutch primary care

**DOI:** 10.1136/bmjph-2024-002057

**Published:** 2025-09-08

**Authors:** Clazinus Veijer, Julia Maria Bes, Christiaan Dolk, Maarten Jacobus Postma, Lisa Aniek de Jong

**Affiliations:** 1 Health Sciences, University Medical Centre Groningen, Groningen, The Netherlands; 2 Pharmacy, PharmacoTherapy, -Epidemiology & -Economics, University of Groningen, Groningen, The Netherlands; 3 NIVEL, Utrecht, The Netherlands; 4 Merck Sharp and Dohme Netherlands, Haarlem, The Netherlands; 5 Economics, University of Groningen Faculty of Economics and Business, Groningen, The Netherlands; 6 Center of Excellence in Higher Education for Pharmaceutical Care Innovation, Universitas Padjadjaran, Bandung, West Java, Indonesia; 7 Pharmocology and Therapy, Airlangga University Faculty of Medicine, Surabaya, Jawa Timur, Indonesia

**Keywords:** human papillomavirus viruses, sexually transmitted diseases, economics, epidemiological monitoring

## Abstract

**Background:**

This study aims to describe the epidemiological trends and estimate the economic burden of genital warts (GW) in Dutch primary care.

**Methods:**

A retrospective, non-interventional, multiyear study (2011–2021) was performed using data from the Nivel Primary Care Database. Changes in incidence by age group, sex and level of urbanisation of individuals with GW and associated healthcare resource use (general practitioner consultations, prescribed medication and referrals) were estimated over the 11-year period. Total annual healthcare costs and cost per incident case were estimated via a bottom-up gross costing approach.

**Results:**

Between 2011 and 2021, GW incidence increased, which was especially seen in men (from 2.0 to 3.5 per 1000 inhabitants) and to a lesser extent in women (from 1.9 to 2.1 per 1000 inhabitants). GW incidence was most common in age group 20–29 years (men: 43.6%; women: 50.7%) and highly urbanised areas. Medication was prescribed in 61.4% of GW cases, and 5.4% of patients with GW were referred to secondary care. Total costs in Dutch primary care increased by 108% from €2.3 million in 2011 to €4.9 million in 2021. The cost per incident case also showed an increasing trend from €72 in 2011 to €99 in 2021. Referrals to secondary care resulted in a 14%–30% increase in total costs.

**Conclusions:**

This study provides novel insights into recent epidemiological trends of GW and its associated costs in Dutch primary care. The incidence of GW increased particularly among men, and the total annual costs of GW in primary care doubled between 2011 and 2021.

**WHAT IS ALREADY KNOWN ON THIS TOPIC**

- Incidence of genital warts (GW) is especially common among young adults and induces substantial healthcare costs.
- Research examining the epidemiology and economic burden of GW is scarce, and outcomes across studies differ substantially.
- Recent details on healthcare resource use (HCRU) and associated costs are essential when planning preventive measures and efficiently allocating healthcare resources.

**WHAT THIS STUDY ADDS**

- This study reveals unique estimates of general practitioner consultations, prescribed medication, referrals and associated costs for patients with GW in the Netherlands.
- It also describes a rising trend in the economic burden of GW in general practitioner care, mainly due to higher incidence among men and higher medical costs.

**HOW THIS STUDY MIGHT AFFECT RESEARCH, PRACTICE OR POLICY**

- This research provides valuable information for the continuous evaluation of both the national immunisation programme against human papillomavirus when integrating GW into its scope and other preventive measures that may counteract the root cause of disease transmission causing GW.
- Furthermore, the HCRU data presented in this study can be used in future cost-effectiveness analyses related to the prevention and treatment of GW.

## Introduction

 Understanding the epidemiological trends and economic burden of a disease is essential when planning preventive measures and efficiently allocating healthcare resources. Especially in the field of infectious diseases, it is useful to evaluate which preventive measures effectively counteract the root cause of disease transmission. One such disease concerns the highly infectious and one of the most common viral sexually transmissible diseases worldwide: genital warts (GW; *Condylomata acuminata*).

GW are benign epithelial skin lesions predominantly caused by an infection with non-oncogenic human papillomavirus (HPV) subtypes 6 and 11.^1^ Worldwide incidence rates range between 100 and 200 new cases per 100 000 general adult population, and peak at the age of 15–29 years.^2^ The majority of cases are asymptomatic and transient, although recurrence of the self-limiting disease after initial clearance or beyond treatment is common.^3^ Treatment options consist of home-based therapy, including self-applied topical treatments podophyllotoxin, imiquimod and sinecatechins, and clinic-based therapy, including chemical treatments and ablative techniques, such as electrotherapy, cryotherapy and laser therapy.^1^ In the Netherlands, the large majority of patient complaints is resolved by the general practitioner (GP), who acts as a gatekeeper for secondary care. Surgical treatment is used in specialised care, that is, secondary care facilities.

Apart from pharmaceutical treatment against GW, a number of preventive therapies exist and are globally used to prevent certain HPV-related diseases. Currently, three prophylactic vaccines are licensed in the European Union, all targeting a different range of HPV types: the bivalent vaccine, Cervarix (targeting HPV types 16/18), the quadrivalent recombinant vaccine, Gardasil (targeting HPV types 6/11/16/18) and the nonavalent vaccine, Gardasil 9 (targeting HPV types 6/11/16/18/31/33/45/52/58). As of 2010, the Netherlands started routine HPV vaccination for girls only using the bivalent vaccine, which does not provide protection against the HPV types 6/11 causing GW.^4^

There are limited data on epidemiology, healthcare resource use (HCRU) and costs of GW in scientific literature. In recent years, a few national studies have described epidemiological trends and have estimated the economic burden of GW.^5^,^11^ Outcome measures differ substantially across these analyses, as well as applied methodologies (eg, Delphi panel vs physician survey), costing methods (bottom-up micro-costing approach vs claims data) and study setting. Although the objectives of the studies are comparable, the methodological variations hamper the generalisability of results to a specific country’s context.

In the Netherlands, the last national study on the economic burden of GW was published two decades ago by van der Meijden *et al*, who retrospectively analysed treatment patterns, resource use and costs per patient.^12^ The study, however, did not report any incidence or prevalence estimates of GW in the Netherlands, and population-level costs were not considered. In recent years, new GW incidence rates are roughly estimated at 46 000 episodes per year in the Dutch population, although details on HCRU and costs are lacking.^13^ The current study aims to describe the epidemiological trends of patients with GW in terms of age, sex and level of urbanisation, and to estimate the HCRU and economic burden of GW in the Netherlands.

## Methodology

### Study design and setting

A retrospective, non-interventional, multiyear study was performed using electronic health record (EHR) data routinely recorded by GPs participating in the Nivel Primary Care Database (Nivel-PCD), to calculate epidemiological trends and the economic burden of GW in the Netherlands between 2011 and 2021.^14^ This study was approved by the relevant governance bodies of Nivel-PCD (nr. NZR00322.032). According to Dutch legislation (article 7:458), obtaining neither informed consent nor approval by a medical ethics committee is obligatory for this kind of observational study.^15^

### Study population

Patients were included if they had been diagnosed by a GP between 2011 and 2021 with *C. acuminata*, based on registration using the International Classification of Primary Care, first edition codes Y76 (men) or X91 (women).^16^ Numbers were extrapolated to the total Dutch population based on age, sex and level of urbanisation. The average number of inhabitants at 1 January of the relevant year was used to calculate each year’s population.^17^

### Patient and public involvement

Patients or the public were not involved in the design, or conduct, or reporting, or dissemination plans of our research.

### Outcome measures and analysis

Epidemiological (ie, incidence) and HCRU data (ie, GP consultations, prescribed drug treatments and referrals to secondary care) were obtained from the Nivel-PCD, comprising routinely recorded data from EHRs of approximately 500 general practices in the Netherlands (10%).^14^ Routinely recorded data from GPs are an important source of information, as every citizen is registered with a GP and GPs act as gatekeepers in the Dutch healthcare system. The economic burden was calculated by multiplying the HCRU data by unit costs. Unit costs were obtained from various publicly available sources and are described in detail below. Costs were calculated in Microsoft Excel (Microsoft, Redmond, Washington, USA). Data visualisations were produced using Microsoft Power BI (V.2.121.903.0).

To avoid false accuracy, estimates that were based on low numbers were replaced by ‘<100’ for absolute and ‘<0.1’ for relative frequencies before being transferred to Excel. In the analysis, these values were imputed by 50 and 0.05 for absolute and relative frequencies, respectively. This was only applicable to data clustered by age group.

### Epidemiology

Epidemiology measures included incident cases of GW reported at the GP. Incidence is defined as the number of new disease episodes of GW presented at the GP in the relevant year and is reported per 1000 inhabitants per year. A disease episode was defined as the period between the date of diagnosis and the time of the last encounter plus half of the duration of the contact-free interval. The contact-free interval was defined as the period in which it is likely a patient will visit the GP again if symptoms persist. For GW, the contact-free interval was 16 weeks.^18^ Incidence was clustered by sex (men; women), age group (six categories: 0–19; 20–29; 30–39; 40–49; 50–69; ≥70 years) and level of urbanisation (cities: areas with >1 500 home addresses/km^2^; towns and semi-dense areas: areas with 1000–1500 home addresses/km^2^; rural areas: areas with <1000 home addresses/km^2^). Age categorisation was dependent on the number of individuals per category, as a sufficiently high level of aggregation is required to prevent users or readers deriving an individual’s data from the sample size.

### Healthcare resource use

GP consultations were clustered by sex, type of consultation and year. As of January 2019, the categorisation of GP consultation types changed to better cover the increasing trends in consultations by phone and email (online supplemental table 1).^19^ Prescribed medication was clustered by sex and year for each type of medication (podophyllotoxin, imiquimod and sinecatechins). Prescriptions were available as the number of prescriptions per incident case with a prescription and as a percentage of the total number of incident cases. Data on referrals to secondary care were available from 2015 to 2021 and were stratified by type of medical specialty per year.

### Economic burden

The cost analysis used a healthcare payer’s perspective, including direct medical costs only. Costs were calculated using a bottom-up gross costing approach, in which absolute HCRU data were multiplied by unit costs.^20^ An overview of unit costs in Euros per year is provided in the online supplemental table 2.

Costs of GP consultations were derived from reference prices per consultation type from the Dutch costing manual for economic evaluations in healthcare.^21^ The costing manual differentiated GP consultations by two types: standard GP consultation and telephone consultation. However, the Nivel-PCD data on the number of consultations were differentiated by more than two types. Therefore, the cost of telephone consultations was used for email consultations, while the cost of a standard consultation was used for all other types of consultations.

The costs of prescribed medication include both the pharmacy’s purchase price of the drug and the pharmacy service charge (prescription fee).^22^ Pharmacy purchase prices were obtained from the *G-Standaard*.^23^ Prices of medication products for GW were selected on the relevant Anatomical Therapeutic Chemical (ATC) classification and included Condylin (D06BB04—podophyllotoxin solution for men), Wartec (D06BB04—podophyllotoxin cream for women^24^), Aldara (D06BB10—imiquimod) and Veregen (D06BB12—sinecatechins, introduced in 2012).^25^ The pharmacy service charge was based on the 2020 tariffs for first-time and standard dispensing as reported by the Foundation for Pharmaceutical Statistics (*Stichting Farmaceutische Kengetallen*).^26^ A weighted prescription fee per year was calculated using the average number of prescriptions per patient. Since costs of GP consultations and prescription fees were not available for each year, these were converted to each specific price year (2011–2021) using the consumer price index from Statistics Netherlands.^27^

Costs of a referral to a secondary care specialist were based on average selling prices of different hospital products within the relevant diagnosis group of the dermatology and venerology specialty (‘*diagnose 21—SOA*’).^28^ Prices were obtained from the *‘Diagnose Behandel Combinatie-informatiesysteem’* dataset (a Dutch variant of the Diagnosis-Related Group (DRG) system) of the Dutch healthcare authority (*Nederlandse Zorgautoriteit*) for secondary care costs.^23 28^ The annual average price per referral visit was calculated by multiplying the share of patients within the diagnosis group by the average selling price per care product that is included in the relevant care product group (‘*Infecties met hoofdzakelijk seksuele overdracht’*).

## Results

### Epidemiology

**Figure 1:**
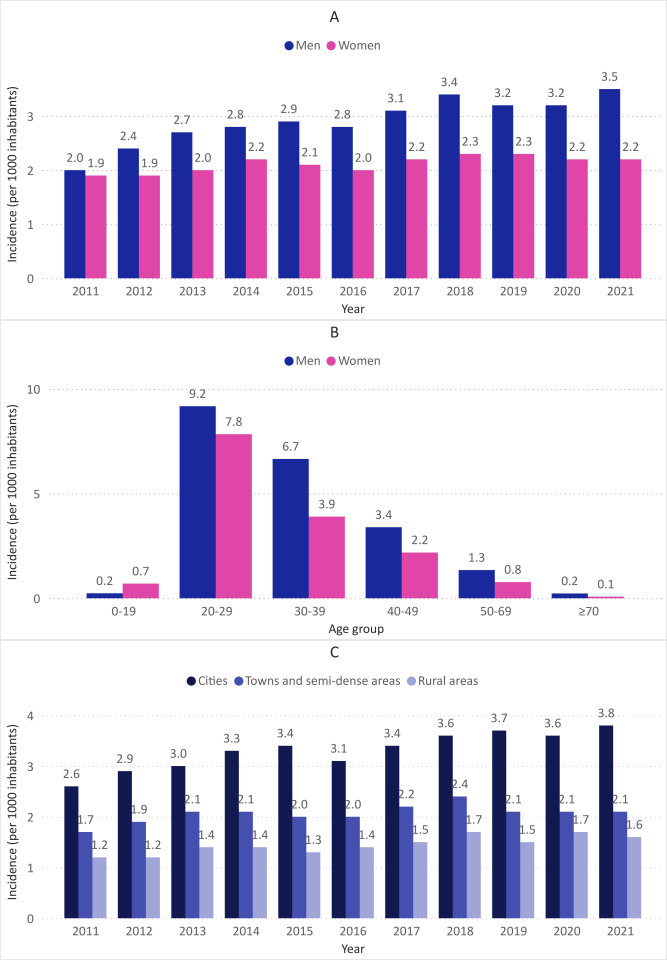
Epidemiology measures of genital warts (GW) based on routinely recorded data from general practitioners (GPs) in the Netherlands between 2011 and 2021. (**A**) Incidence per 1000 inhabitants by sex per year; (**B**) mean incidence per 1000 inhabitants over the period 2011–2021 by sex and age group (reported cases for women in the age group ‘≥70 years’ could not be retrieved because of very low numbers and were imputed by 0.05 per 1000 inhabitants); (**C**) incidence per 1000 inhabitants by level of urbanisation per year.

In the Netherlands, an increase in incidence of GW diagnosed at the GP was observed between 2011 (2.0 per 1000 inhabitants) and 2021 (2.8 per 1000 inhabitants). Over the 11-year period, more men than women were newly diagnosed with GW, and the difference increased steadily over time. Male incidence per 1000 inhabitants increased by 1.5 (2011: 2.0; 2021: 3.5), while female incidence per 1000 inhabitants increased by 0.3 (2011: 1.9; 2021: 2.2) (figure 1A). As shown in figure 1B and online supplemental figure 1, incident cases diagnosed at the GP were highest in the 20–29 age group in both sexes (men: 43.6%; women: 50.7%). Incidence across age groups remained stable over the study period. The proportion of men was higher in all age groups except for the youngest age group (0–19 years). Considering the incidence of GW by level of urbanisation, incident cases were highest in cities, followed by towns/semi-dense areas and rural areas (11-year averages of 3.3, 2.1 and 1.5 per 1000 inhabitants, respectively) (figure 1C). This distribution remained relatively stable over the years.

### Healthcare resource use

**Figure 2:**
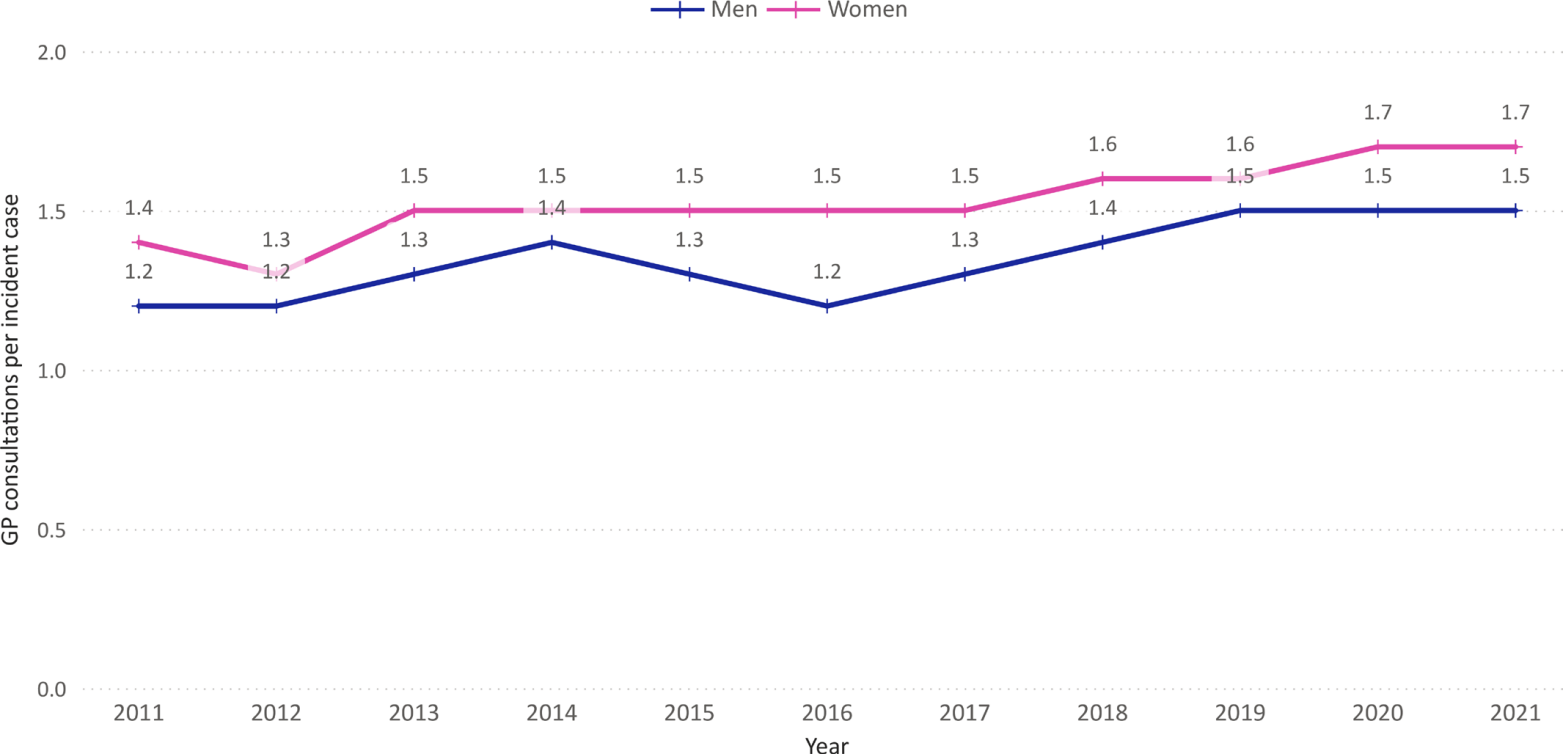
General practitioner (GP) consultations per incident case of genital warts in the Netherlands by sex.

**Figure 3:**
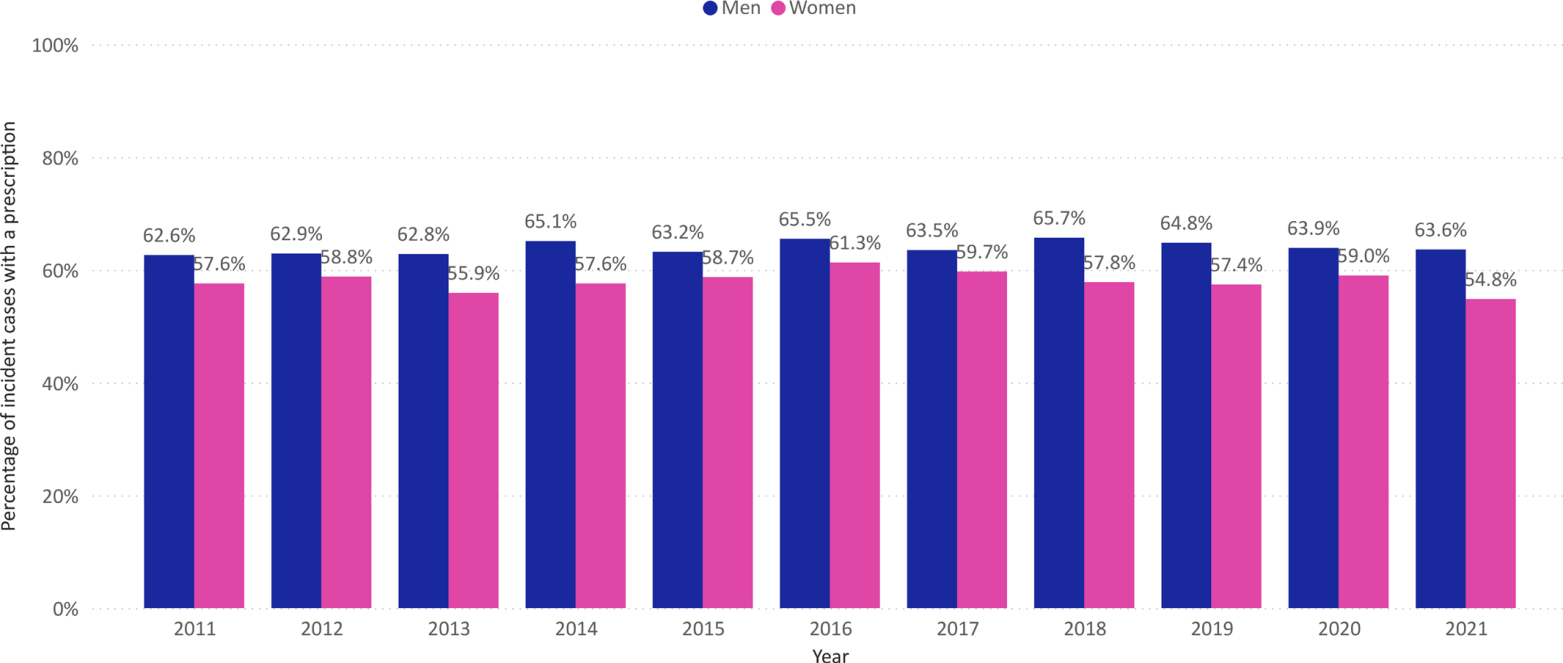
Incident cases of genital warts with a medication prescription as percentage of the total number of incident cases of genital warts in the Netherlands by sex and year.

**Figure 4:**
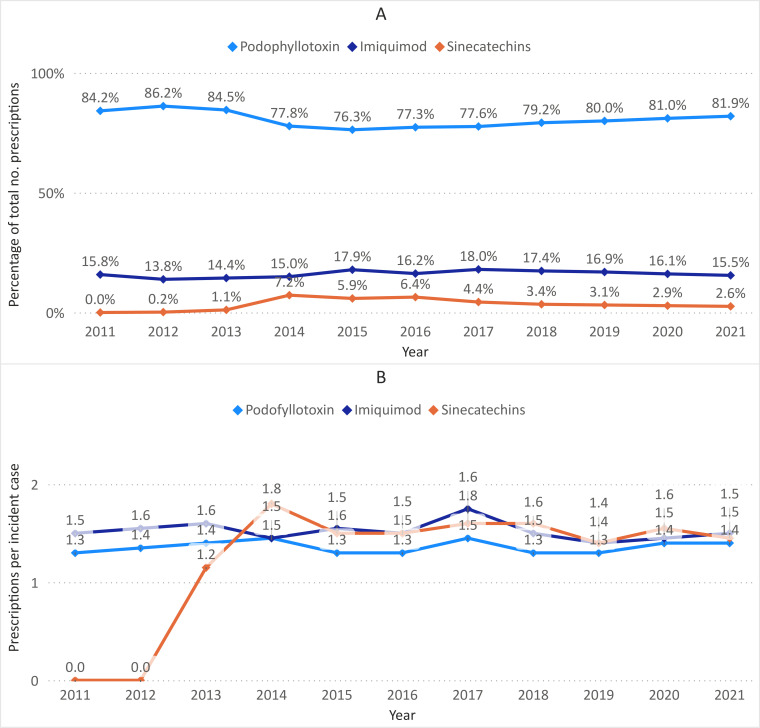
Medication prescriptions for incident cases of genital warts (GW) based on routinely recorded data from general practitioners (GPs) in the Netherlands between 2011 and 2021. (**A**) Prescriptions per type of medication as a percentage of the total number of prescriptions per year for incident cases of GW; (**B**) prescriptions per incident case of GW with a prescription by type of medication.

The number of GP consultations per incident case increased from 1.3 in 2011 to 1.6 in 2021 (average: 1.4; men: 1.3; women: 1.5) (figure 2). On average, 61.4% of GW cases received a prescription for at least one topical treatment (men: 64.0%; women: 58.1%) (figure 3). Of the total number of prescriptions, podophyllotoxin was the most commonly prescribed type of medication (80.6%), followed by imiquimod (16.1%) and sinecatechins (3.4%) (figure 4A). The average number of prescriptions per incident case that received a prescription was 1.4 for podophyllotoxin, 1.5 for imiquimod and 1.5 for sinecatechins (figure 4B). In the period 2015—2021, on average 5.4% of incident cases were referred to a secondary care specialist. The majority of referrals was made to a dermatologist (75.5%), followed by a specialist at the obstetrics and gynaecology department (17.6%) (online supplemental table 3). The number of GW cases observed in the urology department was deemed too low to ensure data privacy and was therefore not included in the current analysis.

### Economic burden

Annual total cost in primary care for GW increased from €2.3 million in 2011 to €4.9 million in 2021. Total costs including costs related to referrals to secondary care increased from €3.9 million in 2015 to €5.8 million in 2021. Costs of GW per year increased by 14%–30% after adding costs of referrals to secondary care. As of 2015, GP consultations made up the majority of total costs (47.4%), followed by prescribed medication costs (36.6%) and referrals to secondary care (16.0%). Costs per incident case also increased (table 1). In primary care, the cost per case increased from €71.80 in 2011 to €98.76 in 2021. The cost per case, including referrals to secondary care, increased from €93.29 in 2015 to €117.40 in 2021.

**Table 1:** Direct medical costs of GW per year and costs per incident case of GW per year for only primary care as well as both primary care and referrals to secondary care

Year	Primary care				Primary care and referrals to secondary care		
	GP consultations (€)	Prescribed medication (€)	Total primary care (€)	Costs per GW case in primary care (€)	Referrals to secondary care (€)	Total including secondary care (€)	Costs per GW case including secondary care (€)
2011	1 297 062	1 052 498	2 349 560	71.80	–	–	–
2012	1 342 027	1 147 382	2 489 409	69.95	–	–	–
2013	1 613 942	1 312 144	2 926 086	74.79	–	–	–
2014	1 816 489	1 732 373	3 548 862	85.12	–	–	–
2015	1 870 226	1 523 172	3 393 399	81.15	507 364	3 900 762	93.29
2016	1 705 341	1 539 553	3 244 895	78.70	670 860	3 915 754	94.98
2017	2 019 494	2 066 432	4 085 927	90.05	821 403	4 907 330	108.15
2018	2 290 774	1 851 250	4 142 024	84.79	1 246 209	5 388 233	110.29
2019	2 627 105	1 754 582	4 381 687	92.72	680 345	5 062 033	107.11
2020	2 734 133	1 897 807	4 631 941	97.54	642 626	5 274 566	111.08
2021	2 995 797	1 901 086	4 896 883	98.76	924 745	5 821 628	117.40

Data on referrals to secondary care were available from 2015 to 2021.

GP, general practitioner; GW, genital warts.

## Discussion

This study is the first analysis of HCRU and associated costs of GW in the Netherlands. Between 2011 and 2021, the incidence of GW in the Netherlands recorded by GPs gradually increased, especially among men. HCRU of GW cases increased accordingly, and total costs increased mainly due to higher incidence and higher medical costs. In general, the results represent a rising trend in the economic burden of GW in primary care. Although a minority of new GW cases were referred by the GP to a secondary care specialist (on average 5.4% of patients), the addition of secondary care costs to primary care costs revealed that these are a substantial part of total costs of GW care. The scope of the current study was limited to describing the epidemiology and estimating HCRU and costs associated with GW, using a large dataset covering ±10% of the total population. No statistical testing for significant trends was conducted, given the size of the dataset.

The increase in incident cases was especially present in men. A recently published report by the Rijksinstituut voor Volksgezondheid en Milieu (RIVM) based on Nivel-PCD data showed similar trends: the number of episodes of GW in men increased from 2017 through 2021, while the number of episodes for women remained relatively stable.^13^ Nonetheless, the reporting rates presented in the RIVM report were extrapolated to the total number of Dutch residents aged 15–64 years, in contrast to our analysis, which extrapolated to the entire Dutch population. As the numerators used in both studies differ, a valid comparison of the number of episodes cannot be made.

As expected, the incidence of GW according to the level of urbanisation was found to be highest in cities. In general, people living in highly urbanised areas tend to be younger and are likely to have more interaction than those living outside these areas. A consistently higher incidence of sexually transmitted infections (STIs) in highly urbanised areas compared with less urbanised areas was also reported in a Nivel report on newly STI-related GP consultations.^29^

Our analysis included data from the first 2 years of the COVID-19 pandemic (2020–2021). Potentially fewer GP consultations due to limited access to healthcare services could have impacted GW-related incidence and costs. However, incidence and costs did not decrease during these 2 years compared with the preceding years in the analysis. In fact, GW incidence increased in 2020 and 2021 for men. As such, the restrictions to healthcare services access during the COVID-19 pandemic were not reflected by the GW-related incidence and costs. Overall, the number of GP contacts in 2021 in the Netherlands did not differ substantially when compared with 2019.^30^ With regard to the diagnosis of STIs in general, both the number of STI-positive episodes and the incidence of the most common STI, chlamydia, was relatively stable during the pandemic.^31^

Medication was prescribed in 61.4% of newly diagnosed GW cases between 2011 and 2021. Prescription rates over the years were consistently higher for men than for women (men: 64.0%; women: 58.1%; at least one prescription per year), which might potentially be explained by the location of the warts. For men, warts are usually situated at the external genital regions, whereas for women, GW more often appear internally, making self-applied treatment less suitable.^32^ Related to this, treatment choice in secondary care may have also been dependent on sex. Men may have been referred more often to the urology specialty for surgical treatment than women. However, the number of GW cases seen in the urology department appeared to be too low to guarantee data privacy and was therefore not included in the current analysis.

Total costs were highest in the final year of the analysis, while the largest annual change in costs was observed in 2017. Apart from a rising number in incidence and subsequent GP consultations, drug costs also increased due to price changes. The average pharmacy purchase price of the podophyllotoxin solution increased by 30.8% within 1 year (2016: €15.38; 2017: €20.13). In 2018, costs of referrals increased by 51.7% compared with 2017, which was mainly due to an enlarged proportion of referrals (online supplemental table 3). A direct reason to explain the higher rate of referrals could not be found. Overall, it is arduous to transfer cost outcomes presented here to other jurisdictions, as differences across countries in inter alia vaccination strategies, healthcare systems and clinical pathways are substantial.^33^

This study is the first analysis of HCRU and associated costs of GW in the Netherlands, and one of the few estimating the economic burden of GW in the European region.^34^,^36^ The results are based on a representative sample of the Dutch population visiting the GP.^14^ The analysis included cost data of the three most relevant resource items in the context of GW. Adding prescription fees to drug costs provided an accurate illustration of the actual expenses made for prescribed treatments.

Nonetheless, the study bears several limitations that should not be neglected. Reported incidence and HCRU and associated costs are surrounded by uncertainties that may have caused an underestimation of the real-world situation. First, data of the Nivel-PCD included reported disease episodes at the GP, leaving GW diagnoses at sexual health centres (SHCs) aside. However, previous research has shown that only around 3% of the GW diagnoses comes from SHCs, indicating that the vast majority of diagnoses is reported at the GP.^13^ Second, many cases may be undetected because of shame or an asymptomatic representation of the disease. A recent study in Catalunya (Spain) estimated that approximately one-fifth of GW cases was not registered, especially for women over the age of 30 years.^37^ Third, costs of secondary care as calculated here are expected to be much higher in actual clinical practice. The number of referrals represents the single fact of a referral being made by a GP to a secondary care specialist, and does not give insight into the number of visits nor the type of treatment given in secondary care. Moreover, the risk of wart recurrence after treatment is substantial (20%–30%), especially among immunocompromised patients, such as HIV-positive individuals.^32^ As such, the actual economic burden of GW in the Netherlands is expected to be even higher than estimated in the current analysis.

On the other hand, several costs may have been overestimated to some extent. The amount of prescribed medications is based on registry data of GPs and may have been overestimated as a consequence of non-dispensing of prescribed medication. A Dutch study showed that approximately 10% of first prescriptions in the dermatological drug class (ATC classification) initiated by a GP was not dispensed at the pharmacy.^38^ Therefore, at least the sum of prescription fees may be lower than calculated here. Furthermore, not all referred patients may have actually visited a secondary care specialist. One study performed in the Netherlands reported referral compliance rates of 77% and 90% for patients referred for the male and female genital system, respectively.^39^ Non-compliance with referrals may result in lower secondary care costs than those estimated in this analysis.

Besides the volume component in the calculation of secondary care costs, the price component should be interpreted with caution as well. Prices pertained only to the care products used within dermatology and venerology specialty, thereby excluding those in the obstetrics and gynaecology specialty. Additionally, the share of patients per care product within the diagnosis group included more than just patients with GW, as the diagnosis group covered STIs more broadly. Moreover, reimbursement claims submitted to health insurers do not accurately reflect the actual expenses made in secondary care.^21^

Further analyses on the economic burden of GW would benefit from more granular data, which would allow for sensitivity analyses on outcome measures and address the uncertainties surrounding current findings. Individual cost data would serve as a valuable input to estimate the cost-effectiveness of HPV-related therapies. The current analysis also lacked data on the procedures performed by the GP as well as the exact treatments applied by a secondary care specialist. Preferably, a micro-costing bottom-up approach should be used, capturing the procedures performed by GPs (to obtain clinic-based as opposed to home-based treatment), indirect cost items (such as travel costs) and HCRU and unit costs in secondary care (in contrast to an average cost per DRG-like payment). Additionally, any future statistical analyses should consider the relationship between clinical relevance and statistical significance.

It is beyond the scope and design of this study to establish correlations between health interventions and the epidemiological data presented. Nonetheless, from an economic point of view, all costs associated with GW care are opportunity costs and could have been prevented if the disease were to be eliminated. The transmission of the HPV causing GW is influenced by sexual behaviour and smoking.^40^ Several strategies exist to reduce the number of GW infections, including education on healthy (sexual) behaviour and vaccination.^41^ A declining incidence of GW is pivotal to abate the economic burden on the Dutch healthcare system.

This study provides novel insights into recent epidemiological trends of GW and its associated HCRU and costs in primary care in the Netherlands. The incidence of GW increased particularly among men, rising from 2.0 per 1000 inhabitants in 2011 to 3.5 per 1000 inhabitants in 2021. Over the same period, the total annual costs of GW in primary care doubled, reaching an estimated €5 million in 2021. The results illustrate the need for effective preventive measures and behavioural awareness aimed at the root cause of GW development.

## Supplementary material

10.1136/bmjph-2024-002057 online supplemental file 1

## References

[1] Federatie Medisch Specialisten (FMS) (2023). Seksueel overdraagbare aandoeningen (SOA) B5. https://richtlijnendatabase.nl/richtlijn/seksueel_overdraagbare_aandoeningen_soa/deel_b_specifieke_soa_s/b5_anogenitale_wratten_condylomata_acuminata.html.

[2] Patel H, Wagner M, Singhal P (2013). Systematic review of the incidence and prevalence of genital warts. BMC Infect Dis.

[3] Centers for Disease Control and Prevention (CDC) (2021). Sexually transmitted infections treatment guidelines. https://www.cdc.gov/std/treatment-guidelines/anogenital-warts.htm.

[4] Woestenberg PJ, Guevara Morel AE, Bogaards JA (2021). Partial Protective Effect of Bivalent Human Papillomavirus 16/18 Vaccination Against Anogenital Warts in a Large Cohort of Dutch Primary Care Patients. Clin Infect Dis.

[5] Berrada M, Holl R, Ndao T (2021). Healthcare resource utilization and costs associated with anogenital warts in Morocco. Infect Agents Cancer.

[6] Prilepskaya VN, Gomberg M, Kothari S (2020). Estimating the Burden of Illness Related to Genital Warts in Russia: A Cross-Sectional Study. *J Health Econ Outcomes Res*.

[7] Lee TS, Kothari-Talwar S, Singhal PK (2017). A cross-sectional study estimating the burden of illness related to genital warts in South Korea. BMJ Open.

[8] Saldarriaga EM, Cárcamo CP, Babigumira JB (2021). The Annual costs of treating genital warts in the Public Healthcare Sector in Peru. BMC Health Serv Res.

[9] Dahlstrom KR, Fu S, Chan W (2018). Medical Care Costs Associated with Genital Warts for Commercially Insured US Patients. Pharmacoeconomics.

[10] Park YJ, Kim JM, Lee BR (2018). Annual prevalence and economic burden of genital warts in Korea: Health Insurance Review and Assessment (HIRA) service data from 2007 to 2015. Epidemiol Infect.

[11] Gylling A, Uusi-Rauva K, Toppila I (2023). The Burden of Genital Warts in Finland: Cross-Sectional Analysis of the Prevalence and Direct Medical Costs in 2018. *Vaccines (Basel*).

[12] van der Meijden WI, Notowicz A, Blog FB (2002). A retrospective analysis of costs and patterns of treatment for external genital warts in The Netherlands. Clin Ther.

[13] Dv W, Visser M, Fv A (2022). Seksueel overdraagbare aandoeningen in Nederland in 2021: Rijksinstituut voor Volksgezondheid en Milieu RIVM.

[14] Nivel (2023). Nivel primary care database. https://www.nivel.nl/en/nivel-zorgregistraties-eerste-lijn/nivel-primary-care-database.

[15] (2023). Dutch civil law, article 7:458. http://www.dutchcivillaw.com/civilcodebook077.htm.

[16] Lamberts H, Wood M (1987). ICPC, International Classification of Primary Care.

[17] Statline CBS The netherlands in figures. https://opendata.cbs.nl/statline/#/CBS/en.

[18] Nielen MMJ, Spronk I, Davids R (2019). Estimating Morbidity Rates Based on Routine Electronic Health Records in Primary Care: Observational Study. JMIR Med Inform.

[19] Dutch healthcare institute (NZa) (2023). Prestatie- en tariefbeschikking huisartsenzorg en multidisciplinaire zorg. https://puc.overheid.nl/nza/doc/PUC_247681_22/1.

[20] Špacírová Z, Epstein D, García-Mochón L (2020). A general framework for classifying costing methods for economic evaluation of health care. Eur J Health Econ.

[21] Zorginstituut Nederland (ZiNL) (2016). Guideline for conducting economic evaluations in healthcare.

[22] Zorginstituut Nederland (ZiNL) (2023). De kosten voor farmaceutische zorg. https://www.farmacotherapeutischkompas.nl/algemeen/kosten.

[23] Z-Index G-Standaard by Z-Index: Essential Data for Dutch Healthcare. https://www.z-index.nl/g-standaard-z-index-essential-data-dutch-healthcare.

[24] Nederlands Huisartsen Genootschap (NHG) NHG Standaard - Het soa-consult.

[25] College ter Beoordeling van Geneesmiddelen (CGB) (2023). Veregen 10%, zalf. https://www.geneesmiddeleninformatiebank.nl/nl/rvg110904.

[26] Stichting Farmaceutische Kengetallen (SFK) (2020). Data en feiten 2020 - het jaar 2019 in cijfers. https://www.sfk.nl/publicaties/data-en-feiten/Dataenfeiten2020.pdf.

[27] Centraal Bureau voor de Statistiek (CBS) Consumer prices; price index 2015=100. https://www.cbs.nl/en-gb/figures/detail/83131eng.

[28] Nederlandse Zorgautoriteit Open data van de nederlandse zorgautoriteit. https://www.opendisdata.nl.

[29] Jaarrapport S (2018). Seksueel overdraagbare aandoeningen (soa). https://www.nivel.nl/nl.

[30] Heins M, Bes J, Weesie Y (2022). Nivel Zorgregistraties Eerste Lijn: jaarcijfers 2021 en trendcijfers 2017-2021.

[31] Visser M Sexually transmitted infections in the netherlands in 20232024.

[32] Gilson R, Nugent D, Werner RN (2020). 2019 IUSTI‐Europe guideline for the management of anogenital warts. Acad Dermatol Venereol.

[33] Raymakers AJN, Sadatsafavi M, Marra F (2012). Economic and humanistic burden of external genital warts. Pharmacoeconomics.

[34] Annemans L, Rémy V, Lamure E (2008). Economic burden associated with the management of cervical cancer, cervical dysplasia and genital warts in Belgium. J Med Econ.

[35] Hillemanns P, Breugelmans JG, Gieseking F (2008). Estimation of the incidence of genital warts and the cost of illness in Germany: a cross-sectional study. BMC Infect Dis.

[36] López N, Torné A, Franco A (2018). Epidemiologic and economic burden of HPV diseases in Spain: implication of additional 5 types from the 9-valent vaccine. *Infect Agent Cancer*.

[37] Moriña D, Fernández-Fontelo A, Cabaña A (2021). Quantifying the under-reporting of uncorrelated longitudal data: the genital warts example. BMC Med Res Methodol.

[38] Hempenius M, Rijken S, Groenwold RHH (2023). Primary nonadherence to drugs prescribed by general practitioners: A Dutch database study. Br J Clin Pharmacol.

[39] van Dijk CE, de Jong JD, Verheij RA (2016). Compliance with referrals to medical specialist care: patient and general practice determinants: a cross-sectional study. BMC Fam Pract.

[40] Tyros G, Mastraftsi S, Gregoriou S (2021). Incidence of anogenital warts: epidemiological risk factors and real-life impact of human papillomavirus vaccination. Int J STD AIDS.

[41] Centers for Disease Control and Prevention (CDC) (2024). How to Prevent STIs. https://www.cdc.gov/sti/prevention/index.html.

